# Efficacy of Pitolisant on the Treatment of Narcolepsy: A Systematic Review

**DOI:** 10.7759/cureus.16095

**Published:** 2021-07-01

**Authors:** Stephanie P Fabara, Juan Fernando Ortiz, Anas Anas Sohail, Jessica Hidalgo, Abbas Altamimi, Belen Tama, Urvish K Patel

**Affiliations:** 1 General Medicine, Universidad Católica de Santiago de Guayaquil, Guayaquil, ECU; 2 Neurology, Universidad San Francisco de Quito, Quito, ECU; 3 Neurology, Larkin Community Hospital, Miami, USA; 4 Medicine, Aureus University School of Medicine, Oranjestad, ABW; 5 Internal Medicine, Universidad San Francisco de Quito, Quito, ECU; 6 Emergency Medicine, Al-Amiri Hospital, Kuwait City, KWT; 7 Medicine, Universidad Católica de Santiago de Guayaquil, Guayaquil, ECU; 8 Public Health and Neurology, Icahn School of Medicine at Mount Sinai, New York, USA

**Keywords:** narcolepsy, pitolisant, modafinil, efficacy, clinical trials

## Abstract

Narcolepsy is characterized by excessive daytime sleepiness (EDS) and cataplexy. Histamine neurons play an important role in enhancing wakefulness. The objective of our study was to evaluate the efficacy of pitolisant, a histamine 3 (H3)-receptor antagonist/inverse agonist, in patients with a high burden of narcolepsy symptoms. We conducted an advanced PubMed search strategy with inclusion and exclusion criteria. The outcome included the Epworth Sleepiness Scale (ESS) and adverse effects frequency. Our primary outcome included the mean ESS score at the endpoint and showed that pitolisant was superior to the placebo, but not non-inferior to modafinil. Adverse effects were less common and shorter in duration in the pitolisant group compared to the modafinil-treated patients. Pitolisant was efficacious in reducing excessive daytime sleepiness and cataplexy compared with placebo, and it was well-tolerated in patients with severe narcolepsy symptoms as compared with modafinil.

## Introduction and background

Narcolepsy is the most common neurological cause of chronic sleepiness [1]. It is characterized by excessive daytime sleepiness and rapid eye movement (REM) sleep dysregulation, causing cataplexy, sleep paralysis, hypnagogic, and hypnopompic hallucinations [2]. The incidence of narcolepsy is 1 in 2,000 individuals [3]. Multiple studies suggest narcolepsy occurs in North America and Europe, with a prevalence of 0.03% - 0.05% [3-4].

There are two types of narcolepsy. Narcolepsy with cataplexy (narcolepsy type I), which is caused by the loss of hypocretin or orexin neurons, and narcolepsy without cataplexy (narcolepsy type II) has normal hypocretin and an unknown etiology. Hypocretin is a neuropeptide produced by neurons in the lateral hypothalamus which promotes wakefulness. Genetic, environmental, and possible autoimmune processes are involved in the pathogenesis of narcolepsy [5-6].

The sleep-wake disturbances in narcolepsy cause several symptoms to a patient’s motor, psychiatric, emotional, cognitive, metabolic, and autonomic functions [7]. Excessive daytime sleepiness (EDS) is a cardinal feature typically causing an inability to stay awake but is also accompanied by difficulties in concentration. Patients experience involuntary, irresistible sleepiness with rapid transitions into sleep, called “sleep attacks” that last from 15 - 20 minutes [8]. A specific symptom of narcolepsy is cataplexy, defined as brief episodes of bilateral loss of muscle tone triggered by sudden emotions in the presence of a normal state of consciousness. The loss of muscle tone seen in cataplexy can manifest as face drooping, eyelid closure, jaw drop, dysarthria, passive tongue protrusion, and bilateral loss of motor control of the extremities [9]. Patients may also experience hallucinations during periods of sleep. Hypnagogic hallucinations occur during sleep onset, while hypnopompic hallucinations occur during awakening. Patients also report sleep paralysis, described as the inability to speak or move any voluntary muscles, usually during awakening [10].

Symptomatic treatment for narcolepsy involves both non-pharmacological and pharmacological approaches. Treatments include counseling, psychosocial guidance, and regular medical follow-up tailored to age, profession, specific lifestyles, and comorbidities [11]. Pitolisant is the last drug to be approved by the Food and Drug Administration (FDA) in 2019 to treat narcolepsy; it showed improvement in previous clinical trials [2]. We conducted a systematic review to pull the data of clinical trials to analyze the Epworth Sleepiness Scale (ESS) and the efficiency of the drug in narcolepsy.

Table 1 shows the five main pharmacological treatments for narcolepsy, dosage, indications, and mechanism of action [2, 12-15].

**Table 1:** Main Treatments of Narcolepsy EDS: excessive daytime sleepiness

Drugs	Dosage	Indication	Mechanism of action
Modafinil	100 - 400 mg	First-line treatment for EDS	Promotes wakefulness by stimulating histamine (HA), norepinephrine (NE), serotonin (5-HT), dopamine (DA), and orexin systems in the brain [12].
Solriamfetol	75 - 150 mg	First-line treatment for EDS	Possible increased activity as a dopamine and norepinephrine reuptake inhibitor [13].
Pitolisant	4.5 - 36.0 mg	First-line treatment for EDS and cataplexy	H3 receptor antagonist/inverse agonist [2].
Methylphenidate	10 - 60 mg	Second-line treatment for EDS	Non-competitively blocks the reuptake of dopamine and noradrenaline into the terminal by blocking dopamine transporter (DAT) and noradrenaline transporter (NAT), increasing levels of dopamine and noradrenaline in the synaptic cleft [14].
Amphetamines	Amphetamine mixed salts: 10 - 60 mg; Dexamphetamine: 10 - 60 mg	Second-line treatment for EDS	Elevates extracellular dopamine (DA) and prolonging DA receptor signaling in the striatum [15].

## Review

Materials and Methods

Protocol

For this systematic review, we used the Preferred Reporting Items for Systematic Reviews and Meta-Analysis (PRISMA) for clinical trials and the Meta-analysis of Observational Studies in Epidemiology (MOOSE) protocol for observational studies. 

Eligibility Criteria and Study Selection

We only included clinical trials (Phase II and above) and observational studies on humans in the last 15 years in the English literature. We excluded all animal studies, studies other than clinical trials and observational studies, and those that did not fulfill the study's outcome. Patients included in the study must have been diagnosed with narcolepsy. After this process, we removed duplicate papers and studies in which the title was not pertinent. 

After screening the studies, we included papers with the following criteria:

1. Patients: Individuals with narcolepsy

2. Intervention: Pitolisant in patients with narcolepsy

3. Comparator: Placebo or control group

4. Outcomes: Epworth Sleepiness Scale

Database and Search Strategy

A review of the literature using PubMed was performed from May 23 to May 30, 2021. The combination of search terms we used was "narcolepsy" and "pitolisant." We used an advanced search strategy with the following terms: (Pitolisant[Title/Abstract]) AND (narcolepsy[Title/Abstract]).

Data Extraction and Analysis

We collected the following information for each study: author and year of publication, methodology, and functional outcomes. Baseline characteristics of the study methods included the number of participants in the treatment, number of participants in the control group, dose route of the administration of the drugs, duration of treatment, and timing when the drugs were given based on the onset of symptoms. Baseline functional outcomes included the Epworth Sleepiness Scale.

Bias Assessment

For assessing bias, we used the Cochrane Collaboration's tool of risk assessment of the clinical trials (RoB 2) and the ROBINS-I tool for the observational studies [16-17].

Results

**Figure 1:**
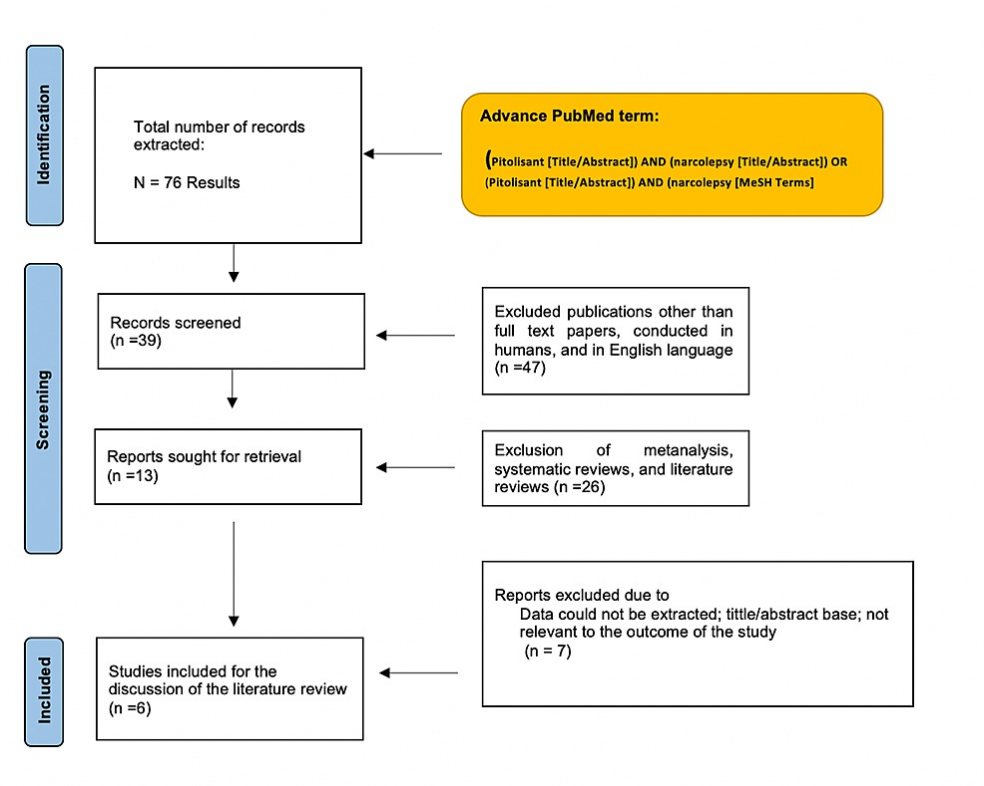
PRISMA flow chart

Figure 1 shows the results of the study using a PRISMA flow chart.

In total, five randomized clinical trials (RCT) and one observational study were considered to be eligible for this systematic review. All of them had a control group. Two studies compared pitolisant, modafinil, and a placebo. One study compared pitolisant vs. placebo, whereas another study compared placebo followed by pitolisant (crossover study). One study compared pitolisant, a placebo, and phentermine, whereas one study compared multiple treatments, including sodium oxybate, mazindol, and methylphenidate.

Table 2 shows the author, year, country, study design, the number of patients in the treatment and the control groups, dose, duration, route of drug administration, and outcomes [18-22].

**Table 2:** Characteristics of Studies Included CI: confidence interval; ESS: Epworth Sleepiness Scale; mg: milligrams; OD: once a day; WCR: weekly cataplexy rate

Author, publication year, reference	Country	Study design	Number of patients in treatment group	Number of patients in control group	Dose, route, and duration	Outcomes
Davis et al., 2021 [18]	Hungary	Post hoc analysis of two randomized studies, placebo-controlled	60	58	Dose up to 35.6 mg orally once daily; seven to eight weeks	ESS score: 19.0 in the pitolisant group and 19.4 in the placebo group. The ESS score was significantly reduced from the baseline value in the pitolisant group (-6.1) compared to the placebo (-2.3; p = < 0.001) after eight weeks.
Dauvilliers et al., 2013 [19]	Switzerland, Germany, France, Hungary, Netherlands	Double-blind randomized, parallel-group controlled trial	32	30 placebo; 33 modafinil	Eight weeks: Week 1: 10 mg OD; Week 2: 20 mg OD; Weeks 3-8: 10, 20, or 40 mg OD; Week 9: oral placebo	ESS score: 12.0 ± 6. 2 in the pitolisant group, 15.6 ± 4.3 in the placebo group, and 11.6 ± 6.0 in the modafinil group. ESS score showed pitolisant was superior to placebo (difference -3.0, 95% CI -5·6 to -0.4; p = 0·024), but not non-inferior to modafinil (difference 0.12, 95% CI -2.5 to 2.7; p = 0·250) after eight weeks.
Setnik et al., 2020 [20]	Canada	Randomized, double-blind, crossover design.	38	38	Single doses of pitolisant 35.6 mg (therapeutic dose), pitolisant 213.6 mg (supratherapeutic dose), phentermine HCl 60 mg, and placebo (oral).	Pitolisant showed significantly lower abuse potential as compared with phentermine. Abuse potential was similar to placebo, which suggests a low risk of abuse for pitolisant.
Lin et al., 2008 [21]	France	Pilot, comparative, sequential placebo-controlled, single-blind, multicenter study.	22	22	Single dose of placebo for one week, followed by oral tiprolisant, 40 mg OD, for one more week (taken in the morning, approximately one hour after awakening)	ESS score: 11.81 ± 6.11 in the treatment group and 16.55 ± 4.86 in the control group. ESS score was reduced from the baseline value of 17.6 by 1.0 with the placebo (p > 0.05) and 5.9 with tiprolisant (p < 0.001).
Inocente et al., 2012 [22]	France	Prospective cohort	4	4	Thirteen months of oral pitolisant, 10 mg in the morning, approximately one hour after awakening OD. If no benefit, dose was increased by 10 mg every week until 40 mg or less in case of adverse effects.	ESS score: 9.5 ± 2.9 in the treatment group and 7 ± 3.5 in the control group. ESS score was reduced from the baseline value of 14.3 ± 1.1 to 9.5 ± 2.9 (p = 0.03) with pitolisant alone and to 7 ± 3.5 when combined with mazindol, methylphenidate, or sodium oxybate, plus modafinil
Szakacs et al., 2020 [23]	Europe (nine different countries)	Randomised, multicenter, double-blind, placebo-controlled trial	54	51	A dose of either 5 mg, 10 mg, or 20 mg of oral pitolisant was used for seven weeks. In the first three weeks, investigators decided on flexible dosing according to the tolerance and efficacy of the drug. There was stable dosing of either 5 mg, 10 mg, 20 mg, or 40 mg in the following four weeks.The primary outcome was the WCR.	The WCR was decreased by 75% in patients in the treatment group vs 38% in the placebo group (p < 0.001). EES decreased by 5.4 in the treatment group vs 1.9 in the placebo group (p < 0.001). Adverse events related to treatment were more prevalent in the pitolisant group as compared to the placebo group with 15 (28%) of 54 vs 6 (12%) of 51; p = 0.048).

The severity of narcolepsy is currently assessed using measures of the ability to stay awake. Because EDS is a frequent complaint in sleep disorders, ESS is often used.

Limitations of the Clinical Trials of Pitolisant and Narcolepsy

The study by Davis et al. used statistical analyses that were specified after the data were seen, with a short-term (seven to eight-week) study duration, and a relatively small sample size in the cataplexy subgroup [18]. In general, post hoc data analysis does not conform to the population or the randomization model of statistical inference, meaning an apparent difference may be a simple coincidence. In addition, the Patient's Global Opinion (PGO) is a nonstandardized rating scale for patients’ impressions of the treatment outcome. As the majority of patients in these studies were diagnosed with narcolepsy type 1, a comparison of EDS responder status between narcolepsy type 1 and narcolepsy type 2 was not possible [18].

The study by Dauvilliers et al. had a short duration which prevents the prediction of whether tolerance can develop on continuation. In addition, the flexible dosage and multiple visits could have affected the efficacy, with less responsive patients being more likely to be titrated to the highest dose [19]. The exclusion of children, severely ill patients, those with unstable comorbidities, and those who refused to potentially receive a placebo during the trial do not allow extrapolation of the efficacy and safety findings to those populations. Furthermore, patients who had previously received modafinil could have been aware that they were receiving it because of its effects, thus negating the masking strategy and affecting the patients' response to treatment. The assessment of withdrawals might be subject to questioning because early withdrawal effects might have been missed if they were not recalled or reported by patients at the later assessment and if the scale used was not sensitive enough since the clinical global impression of change (CGI-C) is a nonvalidated measure in narcolepsy [19].

The study by Setnik et al. had the primary concern of the generalizability of the results. This study involved single-dose administration of pitolisant in a highly controlled setting to a relatively small population of nondependent stimulant users. However, generalizability to other populations or settings may be limited [20].

The results of the study by Lin et al. need to be confirmed in larger populations and by using an optimized dosage and a double-blind design. The hypothesis that H3-receptor inverse agonists alone, or even more in association with modafinil, improve not only excessive daytime sleepiness but also other symptoms of narcolepsy, such as cataplexy, and remains to be scrutinized in appropriate clinical trials [21].

For analysing the bias in the clinical trials, we used the Cochrane Collaboration's risk tool RoB 2 [16]. For analysing the observational studies, we used the Risk of Bias in Non-randomized Studies - of Interventions (ROBINS-I) tool [17]. Table 3 shows the bias in the clinical trials [18-21, 23].

**Table 3:** Bias of the Clinical Trials of Pitolisant Use in Narcolepsy Using the Cochrane Collaboration's Risk of Bias Tool

	Random sequence generation (selection bias)	Allocation concealment (selection bias)	Blinding of participants and personnel (performance bias)	Blinding of outcome assessment	Incomplete outcome data	Selective reporting	Other biases
Davis et al. [18]	Low risk of bias	Low risk of bias	Low risk of bias	Low risk of bias	Low risk of bias	Low risk of bias	Unclear risk of bias
Dauvilliers et al. [19]	Low risk of bias	Low risk of bias	High risk of bias	High risk of bias	Low risk of bias	Low risk of bias	Unclear risk of bias
Setnik et al. [20]	Low risk of bias	Low risk of bias	Low risk of bias	Low risk of bias	Low risk of bias	Low risk of bias	Low risk of bias
Lin et al. [21]	Low risk of bias	Low risk of bias	High risk of bias	High risk of bias	Low risk of bias	Low risk of bias	Unclear risk of bias
Szakacs, et al. [23]	Low risk of bias	Low risk of bias	Low risk of bias	Low risk	Low risk of bias	Low risk of bias	Low risk of bias

The study by Inocente et al. used a small sample size but the result on ESS was already significant, suggesting a large effect size. The improvement was also visible in the Maintenance of Wakefulness Test (MWT), although it did not reach statistical significance owing to the limited sample size [22].

Table 4 shows the analysis of bias of the observational study [22].

**Table 4:** Analysis of the Observational Study Bias

Study	Confounding	Selection bias	Classification of intervention	Deviation from Intervention	Missing data	Measurement of the outcome	Selection of reported result
Inocente et al. [22]	Low	Moderate	Low-risk	Low-risk	Moderate	Low-risk	Low-risk

Discussion

For this systematic review, we used the PRISMA and MOOSE guidelines. The methodology's study included the evaluation of EDS, which was done under the ESS. EDS is the main complaint in sleep disorders and is commonly evaluated using the ESS, which uses eight items to produce a maximal score of 24.

For the present study, we analyzed two main narcolepsy symptoms: EDS and cataplexy, meanwhile other symptoms (e.g., hallucinations or sleep attacks) were not analyzed because they were not well-documented in the trial.

Overall, these clinical trials of pitolisant showed good results in reducing sleepiness. In the clinical trials with pitolisant, modafinil, and placebo, they showed improvement for EDS but were non-inferior to modafinil [19]. Interestingly, three studies showed that the pitolisant has a low abuse potential, compared to regular treatment, since it does not stimulate the nucleus accumbens [20]. One study reported that pitolisant reduced sleepiness, was refractory to all existing previous stimulants, and the ESS score was reduced with the medication combination [22]. Ideally, future studies should be conducted with larger sample sizes for better results.

The Efficacy and Abuse Potential of Pitolisant 

Davis et al. showed that the ESS score was significantly reduced from the baseline value in the pitolisant group (-6.1) compared to the placebo (-2.3; p = < 0.001) [18]. Pitolisant-treated patients had an ESS score reduction of ≥ 3 (69% in the pitolisant group versus 35% in the placebo group, p = 0.001); the final ESS score of ≤ 10 was observed in 36.2% versus 10.5%, respectively (p = 0.005). Headache, nausea, and anxiety were the most common adverse events in the pitolisant-treated patients. Since pitolisant does not increase dopamine release in the nucleus accumbens, it has minimal abuse potential. Pitolisant was efficacious for reducing both EDS and cataplexy, and it also was well tolerated in patients with severe narcolepsy symptoms [18].

The study of Dauvillier et al. compared the ESS score with modafinil, in addition to pitolisant [19]. Reductions were -6.9 (6.2) in the modafinil group, -5.8 (6.2) in the pitolisant group, and -3.4 (4.2) in the placebo group. The ESS decreased at a similar rate in the pitolisant and modafinil group. The ESS score after treatment showed that the pitolisant was superior to placebo; however, pitolisant was not non-inferior to modafinil (difference: 0.12, 95% CI -2.5 to 2.7; p = 0.250). Pitolisant was also well-tolerated compared to modafinil. Regarding adverse effects, one patient had abdominal discomfort with pitolisant, while five experienced abdominal pain, abnormal behaviour, amphetamine-like withdrawal symptoms, lymphoadenopathy, and inner ear disorders with modafinil, showing that pitolisant was better tolerated than modafinil [19].

Setnik et al. evaluated the abuse potential of pitolisant at two different doses (35.6 mg and 213.6 mg) compared to phentermine (a substituted amphetamine) and placebo [20]. Pitolisant produced pharmacodynamic responses that demonstrated significantly lower abuse potential compared with phentermine. Given the public health crisis related to abuse and misuse of prescription drugs, a new treatment with minimal risk of abuse is an important therapeutic option for patients with narcolepsy. The most common adverse events associated with pitolisant were headaches, insomnia, and nausea. Unlike phentermine, pitolisant did not produce any clinically significant increases in blood pressure or heart rate. The authors concluded that pitolisant demonstrated a significantly lower potential for abuse compared with phentermine and an overall profile similar to placebo; this suggests a low risk of abuse for pitolisant [20].

The ESS score in the Lin et al. study showed a reduction of somnolence from a baseline value of 17.6 by 1.0 with the placebo (p = > 0.05) and 5.9 with pitolisant (p = < 0.001), as compared to baseline, appears equivalent to the results obtained after several months of treatment with modafinil [21]. Furthermore, pitolisant requires several days to achieve optimal efficacy, presumably in relationship with the four to five day delay to reach the steady-state. The overall frequency of adverse events was higher during the pitolisant treatment (50% of patients) in comparison to placebo (31.8%). The most frequent adverse events were headache, nausea, and insomnia, experienced mainly during the first three days of treatment. We can say that the pitolisant at the dose of 40 mg per day appears to be efficient in treating EDS of narcoleptic patients and can also be well-tolerated.

In the study of Inocente et al., the ESS score decreased significantly from 14.3 ± 1.1 to 9.5 ± 2.9 (p = 0.03) with pitolisant alone, and even further when combined with mazindol, methylphenidate, or sodium oxybate, plus modafinil (7 ± 3.5) [22]. Adverse effects were minor (insomnia, headache, hot flushes, leg pain, and hallucinations) and transitory, mainly observed during the first week of treatment. The treatment was efficient alone in only one patient, and in three patients, it was necessary to combine it. They concluded that pitolisant could be an alternative treatment that causes few adverse effects [22].

The study by Szakacs et al. improved ESS scores and reduced the weekly cataplexy rate (WCR) in patients with narcolepsy [23]. This was the only study that incorporated patients with high rates of cataplexy attacks. During the study, there was a high degree of "placebo effect" among patients with cataplexy. The effect on cataplexy by modafinil and other stimulants is mainly unknown. Pitolisant presents a solution to reduced cataplexy attacks. However, the mechanism by which pitolisant reduced cataplexy is mainly unknown. The amygdala plays a role in the cataplexy attacks, mainly through gamma-aminobutyric acid (GABA) neuron projections which project to the pontomedullary centers. In this center, GABA neurons mediate the atony in the cataplexy attacks. There is induction of cataplexy in the amygdala, mainly from the histaminergic input of the tuberomammillary nucleus. In this nucleus, there is a high degree of H3 receptors. The H3 inverse agonist of pitolisant on these receptors could potentially be the reason for the reduced frequency in the cataplexy attacks in these patients.

New Direction in the Treatment of Narcolepsy

The symptomatic treatment of narcolepsy has advanced considerably due to the introduction of new effective drugs approved by the European Medicines Agency (EMA) and the Food and Drug Administration (FDA) [7].

Nowadays, there are several drugs used to treat the two main symptoms of narcolepsy: EDS and cataplexy. Modafinil is the first-line treatment for EDS and may have fewer sympathomimetic effects than amphetamines, but it must be used cautiously in people with a history of arrhythmias or heart disease. Methylphenidate and amphetamines are the second-line treatment for EDS but can generate addiction [7].

Solriamfetol (Sunosi™) was approved by the FDA in 2019. Solriamfetol was studied in a double-blind trial of 236 adults with narcolepsy who were randomly assigned one of the three doses of solriamfetol (75, 150, or 300 mg) or a placebo. The ESS score was significantly reduced from the baseline value, -6.4, -5.4, and -3.8 for the 300 mg, 150 mg, and 75 mg doses of solriamfetol, respectively, and -1.6 with the placebo (p < 0.0001) [24]. Solriamfetol has not been compared to or studied in combination with amphetamines, modafinil, or methylphenidate.

FT218 is a once-nightly formulation of sodium oxybate. In March 2020, a multinational, multicentre, double-blind, placebo-control phase III trial was performed to assess the safety and efficacy of FT218 for the treatment of EDS and cataplexy in patients with narcolepsy. Unfortunately, no safety or tolerability findings have been reported in published abstracts on studies in healthy volunteers. To date, this new drug is under review by the FDA before approval [25].

Study Limitations

The present study had several limitations. The analysis was based on a systematic review of four RCTs and one observational study that were identified throughout the research and relied upon the availability and accessibility of the publications.

Further clinical trials are needed to investigate the effectiveness of the pharmacological treatment for narcolepsy in children, pregnant women, patients with comorbidities, and patients who have narcolepsy with no cataplexy. Also, new severity scales, vigilance tests, and patient-reported outcomes are needed.

## Conclusions

Pitolisant is the last drug to be introduced in the market to treat narcolepsy. It has shown improvement in reducing sleepiness and fewer adverse effects than the usual medication. After reviewing the studies, we conclude that pitolisant is superior to placebo and non-inferior to modafinil for improving the EDS. Another advantage of pitolisant is the slow abuse potential that it has. Overall, the drug showed significant efficacy of low abuse potential as compared to other drugs. Overall, the drug showed significant efficacy of low abuse potential as compared to other drugs.
